# Effect of Expressions and SNPs of Candidate Genes on Intramuscular Fat Content in Qinchuan Cattle

**DOI:** 10.3390/ani10081370

**Published:** 2020-08-07

**Authors:** Yaxing Li, Gong Cheng, Takahisa Yamada, Jianfeng Liu, Linsen Zan, Bin Tong

**Affiliations:** 1 The State Key Laboratory of Reproductive Regulation and Breeding of Grassland Livestock, School of Life Sciences, Inner Mongolia University, Hohhot 010070, China; liyaxing1995@163.com; 2 National Beef Cattle Improvement Center, College of Animal Science and Technology, Northwest A&F University, Yangling 712100, China; cg4455@126.com; 3 Department of Agrobiology, Faculty of Agriculture, Niigata University, Niigata 950-2181, Japan; tyamada@agr.niigata-u.ac.jp; 4 Key Laboratory of Animal Genetics, Breeding, and Reproduction, Ministry of Agriculture, National Engineering Laboratory for Animal Breeding, College of Animal Science and Technology, China Agricultural University, Beijing 100193, China; liujf@cau.edu.cn

**Keywords:** association, candidate genes, expression, intramuscular fat content, single nucleotide polymorphism

## Abstract

**Simple Summary:**

Intramuscular fat (IMF), which characterizes the marbling, is a key meat quality trait in the beef industry. In this study, we validated the effect of the expression levels and single nucleotide polymorphisms (SNPs) of *AKIRIN2*, *TTN*, *EDG1*, and *MYBPC1* genes on IMF content in Chinese Qinchuan cattle. The results showed that the expression levels of these genes could affect the IMF content, and the SNP in *EGD1* could affect the IMF content in Qinchuan. The information of this study may be applied to effective marker-assisted selection, to increase the levels of marbling in Qinchuan beef production.

**Abstract:**

Marbling is characterized by the amount and distribution of intramuscular fat (IMF). The *AKIRIN2*, *TTN*, *EDG1*, and *MYBPC1* genes are well-known marbling-related genes, which were first identified in Japanese Black beef cattle. The objectives of this study were to analyze the correlation of the expression levels of these genes in the *longissimus* muscle (LM) with IMF content, and the associations between the single nucleotide polymorphisms (SNPs) in these genes and IMF content in Chinese Qinchuan cattle (n = 350). The association analyses showed that the g.42041062G>T SNP in the *EDG1* gene was significantly associated with IMF content in Qinchuan (*p* < 0.05). Further, the expressions of the *EDG1* and *MYBPC1* were up-regulated (*p* < 0.05) in LM of Qinchuan cattle group with low IMF content. Down-regulations of the *AKIRIN2* and *TTN* genes (*p* < 0.05 and *p* < 0.01, respectively) were observed in the Qinchuan cattle group with high IMF content. These results suggest possible effects of the expression levels of selected genes on IMF content in the LM, and the g.42041062G>T SNP in the *EDG1* gene might be useful as a molecular marker for IMF content in Qinchuan.

## 1. Introduction

Marbling is defined by the amount and distribution of intramuscular fat (IMF) in skeletal muscles, which improves the acceptability, palatability, and tenderness of the meat [1,2,3]. With the rapid development of the economy in China, the consumers’ demands for high quality beef are also growing. Thus, it is necessary to focus on important candidate functional genes and find more effective molecular markers to increase the IMF in Chinese native cattle. Qinchuan cattle, which is one of the five most well-known native yellow cattle breeds in China, have naturally good beef quality. However, the Qinchuan breed exhibits lower marbling than Japanese Black beef (JB) cattle, which could be due to lack of selection for marbling. Therefore, better knowledge of the molecular architecture of IMF content in Chinese native cattle may lead to economic benefits to the beef industry.

The akirin 2 (*AKIRIN2*) gene is located within genomic regions of quantitative trait loci (QTLs) for marbling in JB, Angus and Korean native (KN) cattle [4,5,6]. The titin (*TTN*) gene was found in the genomic regions within QTL for marbling in JB [4]. The endothelial differentiation sphingolipid G-protein-coupled receptor 1 (*EDG1*) gene is located within genomic regions of QTLs for marbling in JB [4], as well as in a Belgian Blue × MARC III (^1^⁄₄ Angus, ^1^⁄₄ Hereford, ^1^⁄₄ Red Poll, ^1^⁄₄ Pinzgauer) developed half-sib family, and a Piedmontese × Angus sire developed half-sib family [7]. Moreover, the myosin binding protein C1 (*MYBPC1*) is included in the genomic region of QTLs for marbling in JB [8], Angus [5], and IMF in a Brangus heifer population [9]. Thus, these genes could be considered important candidate genes for IMF content in beef cattle. Furthermore, the c.*188G>A, g.231054C>T, g.1471620G>T, and g.70014208A>G single nucleotide polymorphisms (SNPs) in the *AKIRIN2*, *TTN*, *EDG1*, and *MYBPC1* genes, respectively, showed associations of these SNPs with marbling in JB [8,10,11,12] and KN [6] cattle. However, the relationships of these candidate genes with marbling, particularly with IMF in Chinese native cattle breed, have not been investigated.

Thus, this study investigated the association of the SNPs in *AKIRIN2*, *TTN*, *EDG1* and *MYBPC1* genes with IMF content in Qinchuan, and evaluated the effects of expression levels of these candidate genes on IMF content in the *longissimus* muscle (LM) of Qinchuan.

## 2. Materials and Methods

### 2.1. DNA Samples and Phenotypes

The DNA samples and phenotypes of 350 Qinchuan adult females (aged 18 to 24 months, and unrelated for at least three generations) were provided from the National Beef Cattle Improvement Center, Northwest A & F University [13,14]. These cattle were reared on the same diets, using commercial standard procedures [14]. The measurement of IMF content was described in the previous reports [13,14]. We utilized biopsies of longissimus muscle at the 12–13th ribs from the Qinchuan adult females at 24 months. After IMF content measurement by ultrasound, the five Qinchuan cattle (low group, LG) with the lowest IMF content (average IMF content was 3.44%, ranging from 2.87 to 4.57%), and five cattle (high group, HG) with the highest IMF content (average IMF content was 8.33%, ranging from 8.26 to 8.44%) were selected for sampling. There was no genetic relationship in the ten Qinchuan cattle. Muscle biopsies were immediately dipped into liquid nitrogen and stored at −80 °C until RNA extraction. The animal care and experiments were conducted according to the Administration of Affairs Concerning Experimental Animals (Ministry of Science and Technology, 2004) China. The protocol was approved on 1 March 2018 by the Institutional Animal Care and Use Ethics Committee of Inner Mongolia University, with the permit number for conducting animal experiments of (IMU-2018-01).

### 2.2. SNP Genotyping by iPLEX MassARRAY

For the population consisting of 350 Qinchuan cattle, the four target SNPs were genotyped with the MassARRAY^®®^ SNP genotyping system (Agena Bioscience, San Diego, CA, USA). PCR and extension primers were designed from sequences containing each target mutation and ~100 upstream and downstream bases with Assay Design Suite (http://agenabio.com/assay-design-suite-20-software), using the default settings. The genotype of each SNP was analyzed using the Sequenom MassARRAY iPLEX platform (Sequenom, San Diego, CA, USA) [15]. The resulting data were analyzed using the MassARRAY Typer 4.0 Analyzer software (Agena Bioscience, San Diego, CA, USA).

### 2.3. Real-Time PCR

For ten samples of the HG and LG, total RNA was isolated from 50 mg of frozen LM samples using the RNeasy Fibrous Tissue kit (QIAGEN GmbH, Hilden, Germany), according to the manufacturer’s instructions. Total RNA was quantified by absorbance at 260 nm, and the integrity of total RNA was checked by agarose gel electrophoresis and ethidium bromide staining of the 28S and 18S bands. Total RNA (2 µg) was reverse-transcribed into cDNA, using an iScript Advanced cDNA Synthesis kit for RT-qPCR (Bio-Rad Laboratories, Hercules, CA, USA), according to the manufacturer’s instructions. Real-time PCR was performed using SsoAdvanced SYBR Green Supermix (Bio-Rad, Hercules, CA, USA). The mRNA expression levels of *AKIRIN2*, *TTN*, *EDG1*, and *MYBPC1* genes in the LM were determined with the CFX Connect Real-time PCR Detection System (Bio-Rad, Hercules, CA, USA), using the mRNA-specific primers, as shown in Table S1. The expression of each gene was normalized against *GAPDH* [16]. The conditions of the real-time PCR reactions were the same as described in our previous report [16]. The relative fold change was calculated using the 2^−∆∆Ct^ calculation [17].

### 2.4. Statistical Analyses

We compared the expression levels of the four genes in the LM between HG and LG by Student’s *t* test. The relationship between different genotypes and IMF content of Qinchuan cattle was analyzed in SPSS 24.0 (SPSS, Inc., Chicago, IL, USA). The statistical linear model for this analysis was the same as in our previous report [14]:

*Y_ijk_* = *μ* + *G_i_* + *A_i_* + *A_k_* + *e_ijk_* (1)

where *Y_ijk_* = trait value per individual, *μ* = overall population mean per trait, *G_i_* = fixed effect associated with genotype, *A_i_* = fixed effect of age (months), *A_k_* was the fixed effect due to the age (years) of dam and *e_ijk_* = standard error. When the number of cattle with a given genotype was less than ten, their associations and effects could not be reliably estimated. Therefore, animals with this genotype were excluded from the analysis. The Bonferroni correction was used to adjust *p* values [14]. The allelic frequency of the g.1471620G>T SNP in *EDG1* was compared between Qinchuan and JB [12] breeds by a χ^2^ test.

## 3. Results and Discussion

### 3.1. Associations between Four SNPs in Candidate Genes and IMF Content in Qinchuan Cattle

The previous studies reported that the c.*188G>A SNP in the *AKIRIN2* gene, the g.231054C>T SNP in the *TTN* gene, and the g.70014208A>G SNP in the *MYBPC1* gene had significant effects on the marbling in the JB [8,10,11] and the KN [6] cattle. However, no significant effect of these SNPs on the IMF content was detected in Qinchuan (Table 1). The JB cattle have been subjected to strong selection for high marbling over the last 50 years [18,19]. On the contrary, there was no strong selection for high marbling trait in Qinchuan. These differences in results of association analysis also could be explained by differences in the genetic background of breed and/or methods of measurement for IMF content in these studies.

For the g.1471620G>T SNP of the *EDG1* gene, the IMF content of the TT homozygotes was significantly higher than that of the GG homozygotes (*p* < 0.05) (Table 1), in agreement with that reported for the same SNP by Yamada et al. [12] in JB, suggesting that the T allele of the g.1471620G>T SNP has an effect on the IMF content and could be considered as a candidate molecular marker for marbling, particularly for IMF content in Qinchuan. In addition, the frequency of IMF content-related T allele of the g.1471620G>T SNP in this experimental Qinchuan population was 0.153, which was significantly lower (*p* < 0.001, by a χ^2^ test) than that in the JB (0.585, [12]). This difference of frequency of the T allele also might be owing to the different selection intensity for high marbling trait between Qinchuan and JB.

### 3.2. Effects of Expression Levels of Four Genes on IMF Content in Longissimus Muscle

**Figure 1:**
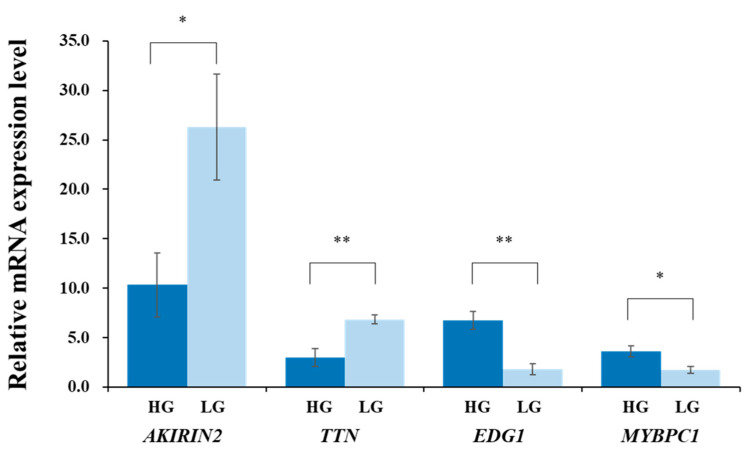
Expression levels of *AKIRIN2*, *TTN*, *EDG1*, and *MYBPC1* genes in *longissimus* muscle of Qinchuan cattle with high and low intramuscular fat content. High group (HG): five Qinchuan cattle with high intramuscular fat (IMF) content. Low group (LG): five Qinchuan cattle with low IMF content. Values are the means ± SE. * *p* < 0.05, ** *p* < 0.01.

The expression levels of the *AKIRIN2*, *TTN*, *EDG1*, and *MYBPC1* genes in the LM of the HG and LG are shown in Figure 1. The expression levels of the *AKIRIN2* and *TTN* genes were significantly higher in the LG than those in the HG (*p* < 0.05 and *p* < 0.01, respectively). Meanwhile, the expression levels of the *EDG1* and *MYBPC1* genes in the LM were significantly higher in the HG than those in the LG (*p* < 0.05 and *p* < 0.05, respectively). Furthermore, the expression patterns of the four genes in the Qinchuan were consistent with previous ddPCR results (using two high-marbled JB and two low-marbled Holstein) [20], also consistent with a qRT-PCR result for the *MYBPC1* expression level in the JB [16], and a qRT-PCR result for the *TTN* expression level in the KN [21]. Thus, these genes could be considered as important candidate genes for IMF content in beef cattle. The three known SNPs in the *AKIRIN2*, *TTN*, and *MYBPC1* genes were not associated with IMF content in this experimental Qinchuan population. There may be breed-specific SNPs of each candidate gene that affect the IMF content in the Qinchuan.

On the basis of the *EDG1* results from association and expression analyses, the *EDG1* gene could be considered as a functional candidate gene for IMF content, and the SNP in *EDG1* might be useful as a molecular marker for IMF content in Qinchuan. However, these results should be confirmed in larger cattle populations in future studies.

In addition, *EDG1* is known to be involved in blood vessel formation [22]. It is likely that the high expression of *EDG1* could increase the proliferation, differentiation or maturation of adipocyte-lineage cells by promoting intramuscular vascularization and then energy provision for the muscle, thereby resulting in increased IMF content. We hypothesize that the SNP has an impact on *EDG1* expression and IMF content by affecting *EDG1* promoter activity or mRNA stability, or both. This hypothesis requires further experimental confirmation.

## 4. Conclusions

In this study, we first showed that the expression levels of the *AKIRIN2*, *TTN*, *EDG1*, and *MYBPC1* genes were associated with IMF content in Qinchuan and the T allele of the g.1471620G>T SNP in the *EDG1* gene was associated with IMF content. This information may be applied to effective marker-assisted selection, to increase the marbling in Chinese Qinchuan beef cattle.

## Figures and Tables

**Table 1:** Association of single nucleotide polymorphisms (SNPs) in the *AKIRIN*2, *TTN*, *EDG1* and *MYBPC1* genes with IMF content in Qinchuan cattle.

Gene	SNP	Genotype (Number)	IMF Content (%)
*AKIRIN2*	c.*188G>A	GG (212)	7.57 ± 0.07
		GA (120)	7.45 ± 0.07
		AA (18)	7.44 ± 0.34
*TTN*	g.231054C>T	CC (257)	7.46 ± 0.06
		CT (87)	7.55 ± 0.10
*EDG1*	g.42041062G>T	GG (255)	7.42 ± 0.07 ^a^
		GT (83)	7.60 ± 0.09
		TT (12)	8.15 ± 0.03 ^b^
*MYBPC1*	g.70014208A>G	GG (291)	7.48 ± 0.06
		GA (53)	7.67 ± 0.09

Note: IMF: intramuscular fat. ^a,b^ means with different letters were significantly different (*p* < 0.05) after Bonferroni correction.

## Supplementary Materials

The following are available online at https://www.mdpi.com/2076-2615/10/8/1370/s1, Table S1: Sequences of real-time PCR primers.
