# Supplementary material for: Effect of Expressions and SNPs of Candidate Genes on Intramuscular Fat Content in Qinchuan Cattle
Source: Animals (Basel). 2020 Aug 7;10(8):1370. doi: 10.3390/ani10081370 (PMC7459438; doi:10.3390/ani10081370)
Supplement: Supplementary file 1 [file animals-10-01370-s001.pdf]

Table S1. Sequences of real-time PCR primers.

Gene	Sequence (5'-3')
AKIRIN2	F: GCGACGATATGGAGAACAGC<br>R: GCCACTGACGAAAGCTTGAA
TTN	F: CCCCATCAGATCCGTCAAGTC<br>R: CTTGCTCCTGGATCCCGTATC
EDG1	F: TCCTGTACTGCAGGATCTACTC<br>R: ACACCAGGAAGTACTCCGTT
MYBPC1	F: CTCCTACTCTTCTGACCGTT<br>R: CACATAGATCCTTGAATCCGTT
GAPDH	F: TGACCCCTTCATTGACCTTCA<br>R: ACCCCAGTGGACTCCACCACAT
